# The management of severe isolated traumatic brain injury in pregnancy: A joint consensus statement from the European Association of Neurosurgical Societies (EANS) and the World Society of Emergency Surgery (WSES)

**DOI:** 10.1016/j.bas.2026.105971

**Published:** 2026-02-19

**Authors:** Edoardo Picetti, Andreas K. Demetriades, Bart Depreitere, Aurelia Peraud, Fausto Catena, Chiara Robba, EANS-WSES TBI in Pregnancy Consensus Group, Bizhan Aarabi, Christian Abel, Fikri M. Abu-Zidan, P. David Adelson, Vanni Agnoletti, Luca Ansaloni, Rocco A. Armonda, Goran Augustin, Rafael Badenes, Zsolt J. Balogh, Daniele Bellantonio, Antonio Belli, Roberto Berretta, Giacomo Bertolini, Alessandro Bertuccio, Walter L. Biffl, Riccardo Boccaletti, Pierre Bouzat, Sérgio Brasil, Etrusca Brogi, Andras Buki, Anselmo Caricato, Fausto Catena, Ana M. Castano-Leon, Carlo Alberto Castioni, Davide Cerasti, CeCe Cheng, Randall M. Chesnut, Mircea Chirica, Alessandro Cipriano, Dario Cirillo, Enrico Cocchi, Federico Coccolini, Raul Coimbra, Carlo Coniglio, Belinda De Simone, Marika De Vito, Cristian Deana, Andreas K. Demetriades, Bart Depreitere, Enrico Fainardi, Maria Grazia Frigo, Alberto Giannini, Daniel A. Godoy, Areg Grigorian, Paolo Gritti, Deepak Gupta, Dubravko Habek, Gregory W.J. Hawryluk, Raimund Helbok, Peter J. Hutchinson, Corrado Iaccarino, Kenji Inaba, Arundhathi Jeyabalan, Ivo Kehayov, Andrew W. Kirkpatrick, Sam Klein, Angelos Kolias, Tommi K. Korhonen, Alfonso Lagares, Giovanni La Motta, Christos Lazaridis, Laura Lippa, Teemu M. Luoto, Deepa Malaiyandi, Nicolò Marchesini, Matthew J. Martin, Niklas Marklund, Geert Meyfroidt, Marina Munari, Jeffry Nahmias, Lena M. Napolitano, Savvas Nicolau, David O. Okonkwo, Luigi Orfeo, Wellingson S. Paiva, Marios C. Papadopoulos, Aurelia Peraud, Ondra Petr, Alessandro Pezzini, Edoardo Picetti, Daniel Pinggera, Rita Pini, Jussi P. Posti, Hemanshu Prabhakar, Zaffer A. Qasim, Antonio Ragusa, Frank A. Rasulo, Sandro Rizoli, Chiara Robba, Frederick B. Rogers, Oreste Romeo, Elham Rostami, Andres M. Rubiano, Carla Bittencourt Rynkowski, Aarti Sarwal, Franco Servadei, Deepak Sharma, Lori Shutter, Ranjit D. Singh, Louis Smith, Philip F. Stahel, Jose I. Suarez, Fabio S. Taccone, Nicole A. Terpolilli, Péter J. Tóth, Christine T. Trankiem, Parmenion P. Tsitsopoulos, Andrew A. Udy, Alex B. Valadka, Thomas A. van Essen, Albert J. Varon, Monica S. Vavilala, Marco Vergano, Walter Videtta, Alexander Younsi, Marcello Zinelli, Tommaso Zoerle, Gianluigi Zona

**Affiliations:** j Department of Neurosurgery, University of Maryland School of Medicine, Baltimore, MD, USA; k Department of Interventional Radiology, John Hunter Hospital, Newcastle, New South Wales, Australia; l Department of Surgery, College of Medicine and Health Sciences, United Arab Emirates University, Al Ain, United Arab Emirates; m Department of Neurosurgery, WVU Rockefeller Neuroscience Institute, West Virginia University, Morgantown, WV, USA; n Anesthesia and Intensive Care Department, Bufalini Hospital-Azienda Unità Sanitaria Locale (AUSL) della Romagna, Cesena, Italy; o General and Emergency Surgery Unit, Fondazione IRCCS Policlinico San Matteo, Pavia, Italy; p University of Pavia, Pavia, Italy; q Department of Neurosurgery, MedStar Georgetown University Hospital, Washington, DC, USA; r Department of Surgery, University Hospital Centre Zagreb, Zagreb, Croatia; s Department of Anesthesiology and Surgical-Trauma Critical Care, Hospital Clínic Universitari, University of Valencia, Valencia, Spain; t Department of Traumatology, John Hunter Hospital and University of Newcastle, Newcastle, NSW, Australia; u Department of Anesthesia and Intensive Care, Maria Cecilia Hospital, GVM International, Cotignola, Italy; v The Department of Neurosurgery, University Hospitals Birmingham NHS Foundation Trust, Birmingham, UK; w Department of Gynecology and Obstetrics, University Hospital of Parma, Parma, Italy; x Department of Neurosurgery, Parma University Hospital, Parma, Italy; y Department of Neurosurgery, St. Antonio and Biagio and Cesare Arrigo Hospital, Neurosurgery Unit, Alessandria, Italy; z Department of Surgery, Scripps Clinic/Scripps Clinic Medical Group, La Jolla, CA, USA; aa Department of Anesthesiology and Intensive Care Medicine, Grenoble Alps University Hospital, Grenoble, France; ab Division of Neurosurgery, Department of Neurology, University of São Paulo Medical School, São Paulo, Brazil; ac Intensive Care Unit, ASST Grande Ospedale Metropolitano Niguarda, Milan, Italy; ad School of Medical Sciences, Faculty of Medicine and Health, Örebro University, Örebro, Sweden; ae Department of Neurosurgery, Region Örebro County, Örebro University Hospital, Örebro, Sweden; af Institute of Anesthesiology and Intensive Care, Catholic University School of Medicine, Rome, Italy; ag Department of Medical and Surgical Sciences, Alma Mater Studiorum University of Bologna, Bologna, Italy; ah Department of General and Emergency Surgery, Bufalini Hospital, AUSL Romagna, Cesena, Italy; ai Servicio de Neurocirugía, Hospital Universitario 12 de Octubre, Facultad de Medicina, Departamento de Cirugía, Universidad Complutense de Madrid, Instituto de Investigación Sanitaria Hospital 12 de Octubre (Imas12), Madrid, Spain; aj IRCCS Istituto delle Scienze Neurologiche di Bologna, UOC Anestesia e Rianimazione, Bologna, Italy; ak Neuroradiology Unit, Parma University Hospital, Parma, Italy; al Department of Obstetrics & Gynecology, Division of Maternal-Fetal Medicine, University of Washington, Seattle, WA, USA; am Department of Neurological Surgery, University of Washington, Seattle, WA, USA; an Department of Digestive and Emergency Surgery of the University Hospital of Grenoble, University Grenoble Alpes, Grenoble, France; ao Emergency Department, Nuovo Santa Chiara Hospital, Azienda Ospedaliero-Universitaria Pisana, Pisa, Italy; ap Department of Neurosciences, Reproductive and Odontostomatological Sciences, University of Naples “Federico II”, Naples, Italy; aq Neonatal and Pediatric Intensive Care Unit, AUSL Romagna, Cesena, Italy; ar General Emergency and Trauma Surgery, Pisa University Hospital, Pisa, Italy; as Division of Trauma and Acute Care Surgery and Comparative Effectiveness and Clinical Outcomes Research Center, Riverside University Health System, Moreno Valley, CA, USA; at Department of Anesthesia, Intensive Care and Prehospital Emergency, Ospedale Maggiore Carlo Alberto Pizzardi, Bologna, Italy; au Department of Emergency and Digestive Minimally Invasive Surgery, Infermi Hospital, AUSL Romagna, Rimini, Italy; av Department of Obstetrics and Gynecology, Maggiore Hospital Carlo Alberto Pizzardi, Bologna, Italy; aw Department of Anesthesia and Intensive Care, Health Integrated Agency of Friuli Centrale, Udine, Italy; ax Department of Neurosurgery, Royal Infirmary of Edinburgh, Edinburgh, UK; ay Department of Neurosciences, KU Leuven, Leuven, Belgium; az Department of Neurosurgery, University Hospitals Leuven, Leuven, Belgium; ba Neuroradiology Unit, Department of Experimental and Clinical Biomedical Sciences, University of Florence, Florence, Italy; bb Obstetric Anesthesia and Resuscitation, Isola Tiberina Hospital - Gemelli Isola, Rome, Italy; bc Unit of Pediatric Anesthesia and Intensive Care, Ospedale dei Bambini - ASST Spedali Civili di Brescia, Brescia, Italy; bd Neurointensive Care, Sanatório Pasteur, Catamarca, Argentina; be Department of Surgery, Division of Trauma, Burns, & Surgical Critical Care, University of California, Irvine, Orange, CA, USA; bf Department of Anesthesia and Critical Care Medicine, Papa Giovanni XXIII Hospital, Bergamo, Italy; bg Department of Neurosurgery, All India Institute of Medical Sciences, New Delhi, India; bh Department of Obstetrics and Gynecology, Clinical Hospital Merkur, Zagreb, Croatia; bi Cleveland Clinic Lerner College of Medicine of Case Western Reserve University School of Medicine, Cleveland, USA; bj Department of Neurology, Kepler University Hospital, Johannes Kepler University Linz, Linz, Austria; bk Clinical Research Institute of Neuroscience, Johannes Kepler University Linz, Kepler University Hospital, Linz, Austria; bl Academic Division of Neurosurgery, Department of Clinical Neurosciences, University of Cambridge, UK; bm Division of Neurosurgery, University Hospital of Modena, Modena, Italy; bn Division of Acute Care Surgery, Department of Surgery, Keck School of Medicine of the University of Southern California, Los Angeles, CA, USA; bo Department of Obstetrics, Gynecology and Reproductive Sciences, University of Pittsburgh School of Medicine, Magee-Womens Hospital, Pittsburgh, PA, USA; bp Department of Neurosurgery, Faculty of Medicine, Medical University of Plovdiv, Plovdiv, Bulgaria; bq General, Acute Care, Abdominal Wall Reconstruction, and Trauma Surgery, Foothills Medical Centre, Calgary, Canada; br Department of Neurosurgery, Jessa Hospital, Hasselt, Belgium; bs Faculty of Medicine and Life Science, Hasselt University, Hasselt, Belgium; bt Department of Neurosurgery, Oulu University Hospital & University of Oulu, Oulu, North Ostrobothnia, Finland; bu Neurosurgery Department, Hospital Universitario Sanitas Blua Valdebebas, Madrid, Spain; bv Division of Neurocritical Care, Department of Neurology, University of Chicago Medical Center, Chicago, IL, USA; bw Department of Neurosurgery, Ospedale Niguarda, Milan, Italy; bx Department of Neurosurgery, Tampere University Hospital and Tampere University, Tampere, Finland; by Division of Neurocritical Care, Department of Neurosurgery, University of Michigan, Ann Arbor, MI, USA; bz Department of Neurosurgery, Azienda Ospedaliera Universitaria Integrata di Verona, Verona, Italy; ca Division of Acute Care Surgery, Department of Surgery, University of Southern California, Los Angeles, CA, USA; cb Department of Clinical Sciences Lund, Neurosurgery, Lund University, Skåne University Hospital, Lund, Sweden; cc Department of Intensive Care Medicine, University Hospitals Leuven, Leuven, Belgium; cd Department of Anesthesia and Intensive Care, University Hospital of Padua, Padua, Italy; ce Department of Surgery, University of Michigan Health System, Ann Arbor, MI, USA; cf Department of Radiology, Vancouver General Hospital, University of British Columbia, Vancouver, Canada; cg Department of Neurological Surgery, University of Pittsburgh, Pittsburgh, PA, USA; ch Neonatology and Neonatal Intensive Care Unit, Isola Tiberina-Gemelli Isola Hospital, Rome, Italy; ci Hospital das Clínicas, School of Medicine, Universidade de São Paulo, Sao Paulo, Brazil; cj Academic Neurosurgery Unit, St. George's, University of London, London, UK; ck Division of Pediatric Neurosurgery, Department of Neurosurgery, University Hospital Ulm, Ulm, Germany; cl Department of Neurosurgery, Medical University Innsbruck, Innsbruck, Austria; cm Department of Medicine and Surgery, University of Parma, Parma, Italy; cn Stroke Care Program, Department of Emergency, Parma University Hospital, Parma, Italy; co Department of Anesthesia and Intensive Care, Parma University Hospital, Parma, Italy; cp Neurocenter, Department of Neurosurgery and Turku Brain Injury Center, Turku University Hospital and University of Turku, Turku, Finland; cq Department of Neuroanesthesiology and Neurocritical Care, All India Institute of Medical Sciences, New Delhi, India; cr Department of Emergency Medicine, Perelman School of Medicine, University of Pennsylvania, Philadelphia, PA, USA; cs Neuroanesthesia, Neurocritical and Post-Operative Care, ASST Spedali Civili University Hospital, Brescia, Italy; ct Department of Surgery, Trauma Surgery, Hamad Medical Corporation, Doha, Qatar; cu IRCCS Policlinico San Martino, Dipartimento di Scienze Chirurgiche Diagnostiche e Integrate, Università di Genova, Genoa, Italy; cv Department of Surgery, Regions Hospital, St. Paul, MN, USA; cw Bronson Trauma Surgery, Kalamazoo, USA; cx Section of Neurosurgery, Department of Medical Sciences, Uppsala University, Uppsala, Sweden; cy Neurosciences Institute, El Bosque University, Bogotá, Colombia; cz Intensive Care Unit, Hospital Cristo Redentor - Porto Alegre (RS), Brazil; da Division of Neurocritical Care, Faculty of Medicine, Virginia Commonwealth University, Richmond, VA, USA; db Global Neurosurgery Programme, Besta Neurological Institute, Milan, Italy; dc Neuroanesthesia & Perioperative Neuroscience, University of Washington, Seattle, WA, USA; dd Department of Critical Care Medicine, Neurology and Neurosurgery, University of Pittsburgh School of Medicine, Pittsburgh, PA, USA; de University Neurosurgical Centre Holland (UNCH), Leiden University Medical Centre, Haaglanden Medical Centre and Haga Teaching Hospital, Department of Neurosurgery, Leiden and The Hague, the Netherlands; df Department of Neuroradiology, MedStar Washington Hospital Center, Washington, DC, USA; dg Rocky Vista University, College of Osteopathic Medicine, Parker, CO, USA; dh Division of Neurosciences Critical Care, Johns Hopkins University School of Medicine, Baltimore, MD, USA; di Department of Intensive Care, Hopital Universitaire de Bruxelles (HUB), Université Libre de Bruxelles (ULB), Brussels, Belgium; dj Department of Neurosurgery, LMU Hospital, Ludwig-Maximilian-University Munich, Munich, Germany; dk Department of Neurosurgery, Medical School, University of Pecs, Pecs, Hungary; dl MedStar Washington Hospital Center, Washington, DC, USA; dm Department of Neurosurgery, Hippokratio General Hospital, Aristotle University of Thessaloniki School of Medicine, Thessaloniki, Greece; dn Department of Intensive Care and Hyperbaric Medicine, The Alfred, Melbourne, VIC, 3004, Australia; do Department of Neurological Surgery, University of Texas Southwestern Medical Center, Dallas, TX, USA; dp Department of Anesthesiology, Perioperative Medicine, and Pain Management, University of Miami Miller School of Medicine/Ryder Trauma Center, Miami, FL, USA; dq Department of Anesthesiology and Pain Medicine, University of Washington, Seattle, WA, USA; dr Department of Anesthesia and Intensive Care, San Giovanni Bosco Hospital, Torino, Italy; ds Department of Intensive Care, Posadas Hospital, Buenos Aires, Argentina; dt Department of Neurosurgery, Heidelberg University Hospital, Heidelberg, Germany; du Department of Emergency Medicine, Parma University Hospital, Parma, Italy; dv Neuroscience Intensive Care Unit, Fondazione IRCCS Ca’ Granda Ospedale Maggiore Policlinico, Milan, Italy; dw Department of Pathophysiology and Transplantation, University of Milan, Milan, Italy; dx Department of Neurosurgery, IRCCS Policlinico San Martino, Genoa, and DiNOGMI, University of Genoa, Italy; a Department of Anesthesia and Intensive Care, Parma University Hospital, Parma, Italy; b Department of Neurosurgery, Royal Infirmary of Edinburgh, Edinburgh, UK; c Department of Neurosurgery, Leiden University Medical Centre, Leiden, the Netherlands; d Department of Neurosciences, KU Leuven, Leuven, Belgium; e Department of Neurosurgery, University Hospitals Leuven, Leuven, Belgium; f Division of Pediatric Neurosurgery, Department of Neurosurgery, University Hospital Ulm, Ulm, Germany; g Department of Medical and Surgical Sciences, Alma Mater Studiorum University of Bologna, Bologna, Italy; h Department of General and Emergency Surgery, Bufalini Hospital, AUSL Romagna, Cesena, Italy; i IRCCS Policlinico San Martino, Dipartimento di Scienze Chirurgiche Diagnostiche e Integrate, Università di Genova, Genoa, Italy

**Keywords:** Severe traumatic brain injury, Pregnancy, Intracranial pressure, Management, Consensus, Outcome

## Abstract

**Introduction:**

Severe traumatic brain injury (TBI) during pregnancy is a rare but challenging condition. There is scarce evidence in this population, and severe TBI management during gestation remains empirical and extrapolated from data on non-pregnant women.

**Material and methods:**

The World Society of Emergency Surgery (WSES) and the European Association of Neurosurgical Societies (EANS) collaborated to establish a multidisciplinary consensus panel of 115 physicians with vast expertise in the management of severe TBI, including cases of pregnant women. A modified Delphi approach was adopted. Two online questionnaires were conducted between February and June 2025. The list of statements (36) was distributed to the panelists to allow voting and to propose any comments and/or changes. The analysis of results was performed by an experienced non-voting methodologist. Statements were classified as strong suggestion, weak suggestion or no suggestion when >85%, 75–85% and <75% of votes were in favor, respectively.

**Results:**

A consensus was reached, generating 36 strong suggestions regarding several important aspects in the care of isolated severe TBI during pregnancy.

**Discussion and conclusions:**

This consensus provides practical suggestions to support a clinician's decision-making in the management of severe isolated TBI during pregnancy in high-income countries. However, these statements are based mainly on expert opinion, and further evidence is required in this field.

## 1 Introduction

Severe traumatic brain injury (TBI) during pregnancy is a major non-obstetrical cause of maternal death, and it is associated with poor maternal and fetal outcomes (Al Fauzi et al., 2023; Leach and Zammit, 2020; Ali et al., 1997; Albini et al., 2023; Santos et al., 2024). To date, there is scarce evidence to guide management in this population, as pregnant women are generally excluded from main studies, and therefore severe TBI management during gestation remains empirical and extrapolated from data on non-pregnant women (Al Fauzi et al., 2023; Jain et al., 2015a; Carney et al., 2017; Hawryluk et al., 2019; Di Filippo et al., 2022; Godoy et al., 2022; Heller et al., 2025). A recent international survey showed great variability in the management of pregnant women with severe TBI, highlighting the lack of robust evidence in this setting (Picetti et al., 2025a). This important topic mandates the development of appropriate guidelines to help clinicians provide tailored treatment and, ultimately, maximize maternal and fetal survival (Heller et al., 2025). Considering the above, the specific aim of this multidisciplinary consensus is to provide suggestions for the management of pregnant women with severe isolated TBI.

## 2 Methods

An international multidisciplinary consensus panel was assembled and composed of neurosurgeons (*n* = 44), anesthesiologists/intensivists/neurointensivists (*n* = 34), acute care surgeons (*n* = 21), maternal-fetal medicine (MFM) specialists (*n* = 5), neuroradiologists (*n* = 5), emergency physicians (*n* = 3), neonatologists (*n* = 2), and neurologists (*n* = 1) with expertise in the management of severe TBI, including cases of pregnant women (Supplementary file 1). We ensured geographical representation and diversity. The methodology was similar to that of previously published consensus recommendations (Picetti et al., 2019, 2023, 2024). Initially, we performed a systematic search with PICO and MESH terms (Supplementary file 2). A comprehensive and structured search was conducted across MEDLINE, Embase, and Scopus (Supplementary file 2). The strategy incorporated Medical Subject Headings terms, vocabulary verified by EMBASE terms, and keywords (“pregnancy” and “traumatic brain injury” and/or “intracranial hypertension” and/or “intracranial pressure” and/or “outcome”) to identify studies published between January 1990 and November 2024, with filters for humans, language (English), and time of publication. Inclusion criteria were studies including pregnant patients with severe TBI, and data available on specific management/targets for intracranial pressure (ICP)/cerebral perfusion pressure (CPP) management and clinical outcomes (safety, efficacy, mortality, and neurological outcome). We excluded editorials, commentaries, letters to the editor, opinion articles, reviews, meeting abstracts, original articles lacking an abstract, case reports, and case series. The systematic search resulted in no studies (only case reports/series) which would not go a proper systematic evaluation with GRADING and risk of bias. We therefore used a pragmatic approach, focusing on a scoping review and using data from non-pregnant population, case reports/series and pregnancy physiology. Following the search, the steering committee (EP, AKD, BD, AP, FC, and CR) generated a list of questions for the panel. Two online questionnaires were conducted between February and June 2025. The list of statements (36) was distributed to the panelists for voting and to provide any comments and/or changes. The analysis of results was performed by a non-voting experienced methodologist (CR). Statements were classified as strong suggestion, weak suggestion or no suggestion when >85%, 75–85% and <75% of votes were in favor, respectively. The consensus was focused on the management of severe isolated TBI in pregnant women admitted to a hospital within a high-income country with the availability of neurosurgical, MFM, and neonatal capabilities. This project was endorsed jointly by the World Society of Emergency Surgery (WSES) and the European Association of Neurosurgical Societies (EANS).

## 3 Results

From the literature search, only case reports/series and articles with very low-level evidence were found. A systematic review, published in February 2025, included 16 articles involving a total of 4112 individuals (mean maternal age, 26.9 years; range, 16-47 years) who experienced TBI during pregnancy (mean gestational age at injury, 24 weeks; range, 3-38 weeks) (Heller et al., 2025). The articles comprised 10 case reports, 2 case series, and 4 cohort studies. The risk of bias assessment indicated moderate to good methodological validity overall, but most articles demonstrated poor-quality evidence. Therefore, the consensus is based mainly on no level of evidence and only on the Delphi process and the resultant expert opinion.

**Fig. 1:**
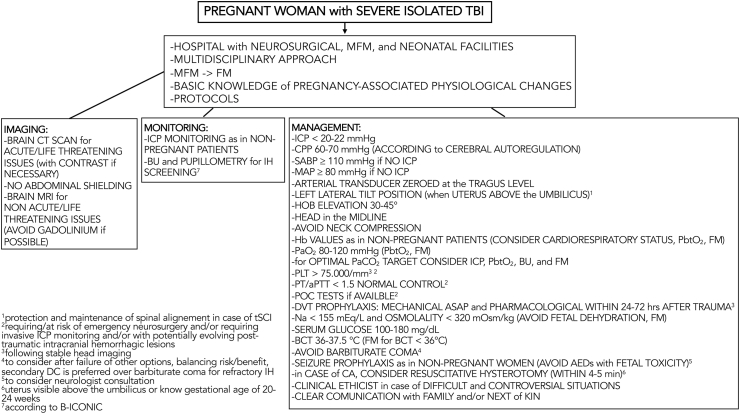
**–** Consensus suggestions. Abbreviations: TBI = traumatic brain injury, MFM = maternal-fetal medicine, FM = fetal monitoring, CT = computed tomography, IH = intracranial hypertension, ICP = intracranial pressure, CPP = cerebral perfusion pressure, SABP = systolic arterial blood pressure, MAP = mean arterial pressure, Hb = hemoglobin, PaO_2_ = arterial partial pressure of oxygen, PaCO_2_ = arterial partial pressure of carbon dioxide, PLT = platelet, PT = prothrombin time, aPTT = activated partial thromboplastin time, POC = point-of-care, MRI = magnetic resonance imaging, P, B-ICONIC = The Brussels consensus for noninvasive ICP monitoring, DVT = deep vein thrombosis, HOB = head of the bed, PbtO_2_ = brain tissue oxygenation, Na = sodium, ASAP = as soon as possible, BCT = body core temperature, hrs = hours, min = minutes, AEDs = antiepileptic drugs, CA = cardiac arrest, tSCI = traumatic spinal cord injury, BU = brain ultrasonography.

The consensus yielded 36 strong suggestions (Table 1, Fig. 1) which are listed below with the associated percentage of agreement.

**Table 1:** **–** List of consensus suggestions.

N.	Suggestion	Level
1	We suggest that pregnant women with severe isolated TBI should be admitted whenever possible to a hospital with neurosurgical, MFM, and neonatal facilities (24/7 availability).	***No evidence, consensus of expert opinion based on clinical experience: “strong”.***
2	We suggest a multidisciplinary approach for the management of pregnant women with severe isolated TBI, thereby involving doctors from different specialties (e.g., neurosurgery, critical care medicine, acute care/trauma surgery, OB&GYN, anesthesiology, neuroradiology/radiology, MFM, emergency medicine, neurology/neurocritical care, and neonatology).	***No evidence, consensus of expert opinion based on clinical experience: “strong”.***
3	We suggest that healthcare professionals involved in the management of pregnant women with severe isolated TBI should be aware of the pregnancy-related physiological changes.	***No evidence, consensus of expert opinion based on clinical experience: “strong”.***
4	We suggest that, in the acute phase and/or life-threatening situations, pregnant women with severe isolated TBI should undergo brain CT scan (with or without intravenous contrast) without delay due to fear of fetal exposure to ionizing radiation/contrasts agents, as in non-pregnant women with severe isolated TBI, and employing radiation-minimizing imaging protocols (standard of new CT scanners).	***No evidence, consensus of expert opinion based on clinical experience: “strong”.***
5	We suggest that the mother's abdomen should not be shielded during a brain CT scan for isolated severe TBI evaluation because the practice of shielding with modern imaging technology can paradoxically increase both total patient and fetal radiation exposure.	***No evidence, consensus of expert opinion based on clinical experience: “strong”.***
6	We suggest that pregnant women with severe isolated TBI at risk for IH (i.e., with radiological signs of increased ICP) should undergo ICP monitoring, as in non-pregnant women with severe isolated TBI.	***No evidence, consensus of expert opinion based on clinical experience: “strong”.***
7	We suggest that the ICP/CPP targets (ICP <20-22 mmHg and CPP = 60-70 mmHg according to the autoregulation test) in pregnant women with severe isolated TBI should be the same as in non-pregnant women with severe isolated TBI.	***No evidence, consensus of expert opinion based on clinical experience: “strong”.***
8	We suggest that, in pregnant women with severe isolated TBI undergoing invasive ABP monitoring, the arterial transducer should be zeroed at the level of the tragus, as in non-pregnant women with severe isolated TBI.	***No evidence, consensus of expert opinion based on clinical experience: “strong”.***
9	We suggest, in pregnant women with severe isolated TBI without ICP/CPP monitoring, that the SABP should be maintained ≥110 mmHg or the MAP ≥80 mmHg, as in non-pregnant women with severe isolated TBI.	***No evidence, consensus of expert opinion based on clinical experience: “strong”.***
10	We suggest that pregnant women with severe isolated TBI should be maintained with the head of the bed elevated at 30°-45° to facilitate brain venous drainage, as in non-pregnant women with severe isolated TBI.	***No evidence, consensus of expert opinion based on clinical experience: “strong”.***
11	We suggest that, in pregnant women with severe isolated TBI, the head should be maintained in the midline, avoiding compression of the neck veins, as in non-pregnant women with severe isolated TBI.	***No evidence, consensus of expert opinion based on clinical experience: “strong”.***
12	We suggest that pregnant women with severe isolated TBI (generally after mid-pregnancy, when the uterus is at or above the umbilicus) should be positioned∗ in left lateral tilt (or left manual displacement of the uterus applied) to alleviate negative hemodynamic effects related to the uterine compression of the inferior vena cava∗∗. ∗Careful positioning (i.e., protection and maintenance of spinal alignment) especially in case of spinal injury. ∗∗Maintaining head elevation, avoiding compression of the neck veins.	***No evidence, consensus of expert opinion based on clinical experience: “strong”.***
13	We suggest, in pregnant women with severe isolated TBI, the use of transfusion thresholds or target Hb levels similar to non-pregnant female severe TBI patients. Transfusion decisions should be based on the pregnant patient's cardiorespiratory status, advanced neuromonitoring (i.e., brain tissue oxygenation), and fetal monitoring.	***No evidence, consensus of expert opinion based on clinical experience: “strong”.***
14	We suggest, in pregnant women with severe isolated TBI, the maintenance of PaO_2_ at 80-120 mmHg (adjusted for altitude). Higher PaO_2_ levels could be used based on advanced neuromonitoring (i.e., brain tissue oxygenation) and fetal monitoring.	***No evidence, consensus of expert opinion based on clinical experience: “strong”.***
15	Although “physiological” respiratory alkalosis occurs during pregnancy, there is no evidence of the effects of low levels of PaCO_2_ on the injured brain of pregnant women with severe isolated TBI. Considering the above, we suggest that the optimal PaCO_2_ target, in this setting, should be based on neurological (e.g., ICP monitoring, brain tissue oxygenation monitoring, brain ultrasonography) and fetal monitoring.	***No evidence, consensus of expert opinion based on clinical experience: “strong”.***
16	We suggest, in pregnant women with severe isolated TBI (requiring/at risk of emergency neurosurgery and/or requiring invasive ICP monitoring and/or with potentially evolving post-traumatic intracranial hemorrhagic lesions), the maintenance of the PLT count >75.000/mm^3^, as in non-pregnant women with severe isolated TBI.	***No evidence, consensus of expert opinion based on clinical experience: “strong”.***
17	We suggest, in pregnant women with severe isolated TBI (requiring/at risk of emergency neurosurgery and/or requiring invasive ICP monitoring and/or with potentially evolving post-traumatic intracranial hemorrhagic lesions) the maintenance of PT/aPTT at a value of <1.5 normal control, as in non-pregnant women with severe isolated TBI.	***No evidence, consensus of expert opinion based on clinical experience: “strong”.***
18	We suggest, in pregnant women with severe isolated TBI (requiring/at risk of emergency neurosurgery and/or requiring invasive ICP monitoring and/or with potentially evolving post-traumatic intracranial hemorrhagic lesions), if available, the utilization of POC tests (e.g., TEG and ROTEM) to assess and optimize coagulation function, as in non-pregnant women with severe isolated TBI.	***No evidence, consensus of expert opinion based on clinical experience: “strong”.***
19	In non-pregnant women with severe isolated TBI requiring osmotherapy for ICP control, the serum sodium levels should be maintained <155 mEq/L and the osmolality <320 mOsm/kg. In case of pregnancy, we suggest caution to avoid fetal dehydration∗. ∗Fetal dehydration can negatively impact fetal well-being and may be detected through fetal monitoring (maternal dehydration can lower the amniotic fluid index and, in extreme cases, can induce decelerations and changes in fetal heart rate variability).	***No evidence, consensus of expert opinion based on clinical experience: “strong”.***
20	We suggest, in pregnant women with severe isolated TBI, the maintenance of serum glucose levels between 100 and 180 mg/dL.	***No evidence, consensus of expert opinion based on clinical experience: strong.***
21	We suggest, in pregnant women with severe isolated TBI, in the absence of possibilities to target the underlying pathophysiologic mechanism of IH, a stepwise approach to the management of increased ICP, as in non-pregnant women with severe isolated TBI. This stepwise approach allows the level of therapy to be increased gradually (step by step), reserving more aggressive interventions (which are generally associated with greater risks/adverse effects) for situations in which no response is observed.	***No evidence, consensus of expert opinion based on clinical experience: “strong”.***
22	We suggest, in pregnant women with severe isolated TBI at risk of IH/cerebral ischemia, the maintenance of the core body temperature between 36 and 37.5 °C, as in non-pregnant women with severe isolated TBI.	***No evidence, consensus of expert opinion based on clinical experience: “strong”.***
23	In pregnant women with severe isolated TBI, we suggest that barbiturate coma should be avoided if possible∗ as third-tier therapy considering its potential maternal and fetal side effects. ∗It should be considered if alternative treatment options are ineffective, balancing maternal-fetal risk/benefit, and as salvage therapy for the mother in absence of other alternatives. Secondary DC is preferred over barbiturate coma for refractory IH.	***No evidence, consensus of expert opinion based on clinical experience: “strong”.***
24	Utilizing a targeted temperature management strategy for ICP control in pregnant women with severe isolated TBI, we suggest that the body core temperature should not be maintained at a level <36 °C to avoid fetal bradycardia. Lower levels could be utilized cautiously according to fetal monitoring.	***No evidence, consensus of expert opinion based on clinical experience: “strong”.***
25	We suggest that seizure prophylaxis should be utilized in pregnant women with severe isolated TBI as in non-pregnant women with severe isolated TBI.	***No evidence, consensus of expert opinion based on clinical experience: “strong”.***
26	In pregnant women with severe isolated TBI with seizures, we suggest that antiepileptic drugs with known fetal toxicity (e.g., valproate, phenobarbital, phenytoin, etc.) should be avoided, particularly during the first trimester. In this regard, neurologist consultation is advisable.	***No evidence, consensus of expert opinion based on clinical experience: “strong”.***
27	In pregnant women with severe isolated TBI who are not in the acute phase and/or life-threatening situations, we suggest that non-contrast∗ brain MRI should be considered for intracranial diagnostic needs, rather than brain CT scan. ∗Gadolinium-based contrast agents are to be avoided in pregnancy unless there is no alternative study and delaying imaging until the post-partum period would result in direct harm to mother or fetus.	***No evidence, consensus of expert opinion based on clinical experience: “strong”.***
28	We suggest brain ultrasonography∗ [i.e., optic nerve sheath diameter (ONSD), transcranial Doppler (TCD) cerebral blood flow velocity analysis, etc.], if available, in the presence of skilled operators and in combination with clinical and radiological parameters, as a useful screening non-invasive tool for detecting IH in pregnant women with severe isolated TBI∗∗. ∗According to B-ICONIC with at least two different modalities (i.e., NPi from automated pupillometer, PI from TCD/TCCD, nICP formula from TCD/TCCD, and ONSD). ∗∗This technology is not intended as a substitute for invasive ICP monitoring.	***No evidence, consensus of expert opinion based on clinical experience: “strong”.***
29	We suggest automated pupillometry∗, if available, in combination with clinical and radiological parameters, as a useful non-invasive screening tool for detecting IH in pregnant women with severe isolated TBI∗∗. ∗According to B-ICONIC with at least two different modalities (i.e., NPi from automated pupillometer, PI from TCD/TCCD, nICP formula from TCD/TCCD, and ONSD) and considering limitations of pupillometry. ∗∗This technology is not intended as a substitute for invasive ICP monitoring.	***No evidence, consensus of expert opinion based on clinical experience: “strong”.***
30	We suggest that a MFM specialist should be immediately involved in the care of pregnant women with severe isolated TBI to assess fetal viability and well-being, as well as the most appropriate form of fetal monitoring (i.e., ultrasonography, fetal heart rate monitoring).	***No evidence, consensus of expert opinion based on clinical experience: “strong”.***
31	We suggest that intermittent pneumatic compression devices (if available and feasible) should be utilized as soon as possible for DVT prophylaxis in pregnant women with severe isolated TBI.	***No evidence, consensus of expert opinion based on clinical experience: “strong”.***
32	We suggest considering the initiation of pharmacologic DVT prophylaxis within 24-72 hours of injury following stable head imaging in pregnant women with severe isolated TBI. In this regard, a multidisciplinary consultation (e.g., neurosurgeon, MFM specialist, etc.) is advisable.	***No evidence, consensus of expert opinion based on clinical experience: “strong”.***
33	In pregnant women, when the uterus is visible above the umbilicus or known gestational age of 20-24 weeks, with severe isolated TBI suffering a cardiac arrest, we suggest that perimortem caesarean section (also known as resuscitative hysterotomy) should be considered as soon as possible (ideally, within no more than 4-5 minutes).	***No evidence, consensus of expert opinion based on clinical experience: “strong”.***
34	For difficult and controversial situations in the management of pregnant women with severe isolated TBI (e.g., timing of emergency neurosurgery, timing/modality of delivery, abortion, maternal brain death and pregnancy progression, etc.), we suggest a multidisciplinary approach, with the possible aid of a clinical ethicist, to optimize decision-making in this challenging scenario.	***No evidence, consensus of expert opinion based on clinical experience: “strong”.***
35	For difficult and controversial situations in the management of pregnant women with severe isolated TBI (e.g., timing of emergency neurosurgery, timing/modality of delivery, termination of pregnancy, maternal brain death and pregnancy progression, etc.), we suggest a clear communication strategy with the family or next of kin.	***No evidence, consensus of expert opinion based on clinical experience: “strong”.***
36	We suggest that specific hospital protocols, based on the best available evidence and literature, regarding the management of pregnant women with severe isolated TBI, should be encouraged and implemented to optimize maternal and fetal outcomes.	***No evidence, consensus of expert opinion based on clinical experience: “strong”.***

Abbreviations: TBI = traumatic brain injury, MFM = maternal-fetal medicine, OB&GYN = obstetrics/gynecology, CT = computed tomography, IH = intracranial hypertension, ICP = intracranial pressure, CPP = cerebral perfusion pressure, ABP = arterial blood pressure, SABP = systolic arterial blood pressure, MAP = mean arterial pressure, Hb = hemoglobin, PaO_2_ = arterial partial pressure of oxygen, PaCO_2_ = arterial partial pressure of carbon dioxide, PLTs = platelets, PT = prothrombin time, aPTT = activated partial thromboplastin time, POC = point-of-care, TEG = thromboelastography, ROTEM = rotational thromboelastometry, MRI = magnetic resonance imaging, ONSD = optic nerve sheath diameter, nICP = noninvasive ICP, B-ICONIC = The Brussels consensus for nICP monitoring, NPi = neurological pupillary index, PI = pulsatility index, TCD = transcranial Doppler, TCCD = transcranial color coded Doppler, DVT = deep vein thrombosis, DC = decompressive craniectomy.

### 3.1 Suggestion 1

We suggest that pregnant women with severe isolated TBI should be admitted whenever possible to a hospital with neurosurgical, MFM, and neonatal facilities (24/7 availability) (agreement 98.2%, strong suggestion).

### 3.2 Suggestion 2

We suggest a multidisciplinary approach for the management of pregnant women with severe isolated TBI, thereby involving doctors from different specialties [e.g., neurosurgery, critical care medicine, acute care/trauma surgery, obstetrics/gynecology (OB&GYN), anesthesiology, neuroradiology/radiology, MFM, emergency medicine, neurology/neurocritical care, and neonatology] (agreement 99.1%, strong suggestion).

### 3.3 Suggestion 3

We suggest that healthcare professionals involved in the management of pregnant women with severe isolated TBI should be aware of the pregnancy-related physiological changes (agreement 99.1%, strong suggestion).

### 3.4 Suggestion 4

We suggest that, in the acute phase and/or life-threatening situations, pregnant women with severe isolated TBI should undergo brain computed tomography (CT) scan (with or without intravenous contrast) without delay due to fear of fetal exposure to ionizing radiation/contrasts agents, as in non-pregnant women with severe isolated TBI, and employing radiation-minimizing imaging protocols (standard of new CT scanners) (agreement 95.6%, strong suggestion).

### 3.5 Suggestion 5

We suggest that the mother's abdomen should not be shielded during a brain CT scan for isolated severe TBI evaluation because the practice of shielding with modern imaging technology can paradoxically increase both total patient and fetal radiation exposure (agreement 91.2%, strong suggestion).

### 3.6 Suggestion 6

We suggest that pregnant women with severe isolated TBI at risk for intracranial hypertension (IH) (i.e., with radiological signs of increased ICP) should undergo ICP monitoring, as in non-pregnant women with severe isolated TBI (agreement 100%, strong suggestion).

### 3.7 Suggestion 7

We suggest that the ICP/CPP targets (ICP <20-22 mmHg and CPP = 60-70 mmHg according to the autoregulation test) in pregnant women with severe isolated TBI should be the same as in non-pregnant women with severe isolated TBI (agreement 96.5%, strong suggestion).

### 3.8 Suggestion 8

We suggest that, in pregnant women with severe isolated TBI undergoing invasive arterial blood pressure (ABP) monitoring, the arterial transducer should be zeroed at the level of the tragus, as in non-pregnant women with severe isolated TBI (agreement 95.6%, strong suggestion).

### 3.9 Suggestion 9

We suggest, in pregnant women with severe isolated TBI without ICP/CPP monitoring, that the systolic ABP (SABP) should be maintained ≥110 mmHg or the mean arterial pressure (MAP) ≥ 80 mmHg, as in non-pregnant women with severe isolated TBI (agreement 91.2%, strong suggestion).

### 3.10 Suggestion 10

We suggest that pregnant women with severe isolated TBI should be maintained with the head of the bed elevated at 30°-45° to facilitate brain venous drainage, as in non-pregnant women with severe isolated TBI (agreement 98.2%, strong suggestion).

### 3.11 Suggestion 11

We suggest that, in pregnant women with severe isolated TBI, the head should be maintained in the midline, avoiding compression of the neck veins, as in non-pregnant women with severe isolated TBI (agreement 100%, strong suggestion).

### 3.12 Suggestion 12

We suggest that pregnant women with severe isolated TBI (generally after mid-pregnancy, when the uterus is at or above the umbilicus) should be positioned∗ in left lateral tilt (or left manual displacement of the uterus applied) to alleviate negative hemodynamic effects related to the uterine compression of the inferior vena cava∗∗ (agreement 97.4%, strong suggestion).

∗Careful positioning (i.e., protection and maintenance of spinal alignment) especially in case of spinal injury.

∗∗Maintaining head elevation, avoiding compression of the neck veins.

### 3.13 Suggestion 13

We suggest, in pregnant women with severe isolated TBI, the use of transfusion thresholds or target hemoglobin (Hb) levels similar to non-pregnant female severe TBI patients. Transfusion decisions should be based on the pregnant patient's cardiorespiratory status, advanced neuromonitoring (i.e., brain tissue oxygenation), and fetal monitoring (agreement 97.4%, strong suggestion).

### 3.14 Suggestion 14

We suggest, in pregnant women with severe isolated TBI, the maintenance of arterial partial pressure of oxygen (PaO_2_) at 80-120 mmHg (adjusted for altitude). Higher PaO_2_ levels could be used based on advanced neuromonitoring (i.e., brain tissue oxygenation) and fetal monitoring (agreement 99.1%, strong suggestion).

### 3.15 Suggestion 15

Although “physiological” respiratory alkalosis occurs during pregnancy, there is no evidence of the effects of low levels of arterial partial pressure of carbon dioxide (PaCO_2_) on the injured brain of pregnant women with severe isolated TBI. Considering the above, we suggest that the optimal PaCO_2_ target, in this setting, should be based on neurological (e.g., ICP monitoring, brain tissue oxygenation monitoring, brain ultrasonography) and fetal monitoring (agreement 96.5%, strong suggestion).

### 3.16 Suggestion 16

We suggest, in pregnant women with severe isolated TBI (requiring/at risk of emergency neurosurgery and/or requiring invasive ICP monitoring and/or with potentially evolving post-traumatic intracranial hemorrhagic lesions), the maintenance of the platelet (PLT) count >75.000/mm^3^, as in non-pregnant women with severe isolated TBI (agreement 92.1%, strong suggestion).

### 3.17 Suggestion 17

We suggest, in pregnant women with severe isolated TBI (requiring/at risk of emergency neurosurgery and/or requiring invasive ICP monitoring and/or with potentially evolving post-traumatic intracranial hemorrhagic lesions) the maintenance of prothrombin time (PT)/activated partial thromboplastin time (aPTT) at a value of <1.5 normal control, as in non-pregnant women with severe isolated TBI (agreement 96.5%, strong suggestion).

### 3.18 Suggestion 18

We suggest, in pregnant women with severe isolated TBI (requiring/at risk of emergency neurosurgery and/or requiring invasive ICP monitoring and/or with potentially evolving post-traumatic intracranial hemorrhagic lesions), if available, the utilization of point-of-care (POC) tests [e.g., thromboelastography (TEG) and rotational thromboelastometry ROTEM] to assess and optimize coagulation function, as in non-pregnant women with severe isolated TBI (agreement 96.5%, strong suggestion).

### 3.19 Suggestion 19

In non-pregnant women with severe isolated TBI requiring osmotherapy for ICP control, the serum sodium levels should be maintained <155 mEq/L and the osmolality <320 mOsm/kg. In case of pregnancy, we suggest caution to avoid fetal dehydration∗.

∗Fetal dehydration can negatively impact fetal well-being and may be detected through fetal monitoring (maternal dehydration can lower the amniotic fluid index and, in extreme cases, can induce decelerations and changes in fetal heart rate variability) (agreement 97.4%, strong suggestion).

### 3.20 Suggestion 20

We suggest, in pregnant women with severe isolated TBI, the maintenance of serum glucose levels between 100 and 180 mg/dL (agreement 93%, strong suggestion).

### 3.21 Suggestion 21

We suggest, in pregnant women with severe isolated TBI, in the absence of possibilities to target the underlying pathophysiologic mechanism of IH, a stepwise approach to the management of increased ICP, as in non-pregnant women with severe isolated TBI. This stepwise approach allows the level of therapy to be increased gradually (step by step), reserving more aggressive interventions (which are generally associated with greater risks/adverse effects) for situations in which no response is observed (agreement 99.1%, strong suggestion).

### 3.22 Suggestion 22

We suggest, in pregnant women with severe isolated TBI at risk of IH/cerebral ischemia, the maintenance of the core body temperature between 36 and 37.5 °C, as in non-pregnant women with severe isolated TBI (agreement 96.5%, strong suggestion).

### 3.23 Suggestion 23

In pregnant women with severe isolated TBI, we suggest that barbiturate coma should be avoided if possible∗ as third-tier therapy considering its potential maternal and fetal side effects (agreement 97.4%, strong suggestion).

∗It should be considered if alternative treatment options are ineffective, balancing maternal-fetal risk/benefit, and as salvage therapy for the mother in absence of other alternatives. Secondary decompressive craniectomy (DC) is preferred over barbiturate coma for refractory IH.

### 3.24 Suggestion 24

Utilizing a targeted temperature management strategy for ICP control in pregnant women with severe isolated TBI, we suggest that the body core temperature should not be maintained at a level <36 °C to avoid fetal bradycardia. Lower levels could be utilized cautiously according to fetal monitoring (agreement 86.8%, strong suggestion).

### 3.25 Suggestion 25

We suggest that seizure prophylaxis should be utilized in pregnant women with severe isolated TBI as in non-pregnant women with severe isolated TBI (agreement 90.4%, strong suggestion).

### 3.26 Suggestion 26

In pregnant women with severe isolated TBI with seizures, we suggest that antiepileptic drugs with known fetal toxicity (e.g., valproate, phenobarbital, phenytoin, etc.) should be avoided, particularly during the first trimester. In this regard, neurologist consultation is advisable (agreement 97.4%, strong suggestion).

### 3.27 Suggestion 27

In pregnant women with severe isolated TBI who are not in the acute phase and/or life-threatening situations, we suggest that non-contrast∗ brain magnetic resonance imaging (MRI) should be considered for intracranial diagnostic needs, rather than brain CT scan (agreement 93.9%, strong suggestion).

∗Gadolinium-based contrast agents are to be avoided in pregnancy unless there is no alternative study and delaying imaging until the post-partum period would result in direct harm to mother or fetus.

### 3.28 Suggestion 28

We suggest brain ultrasonography∗ [i.e., optic nerve sheath diameter (ONSD), transcranial Doppler (TCD) cerebral blood flow velocity analysis, etc.], if available, in the presence of skilled operators and in combination with clinical and radiological parameters, as a useful screening non-invasive tool for detecting IH in pregnant women with severe isolated TBI∗∗ (agreement 88.6%, strong suggestion).

∗According to B-ICONIC [The Brussels consensus for noninvasive ICP (nICP) monitoring] with at least two different modalities [i.e., neurological pupillary index (NPi) from automated pupillometer, pulsatility index (PI) from TCD/transcranial color coded Doppler (TCCD), nICP formula from TCD/TCCD, and ONSD].

∗∗This technology is not intended as a substitute for invasive ICP monitoring.

### 3.29 Suggestion 29

We suggest automated pupillometry∗, if available, in combination with clinical and radiological parameters, as a useful non-invasive screening tool for detecting IH in pregnant women with severe isolated TBI∗∗ (agreement 92.1%, strong suggestion).

∗According to B-ICONIC with at least two different modalities (i.e., NPi from automated pupillometer, PI from TCD/TCCD, nICP formula from TCD/TCCD, and ONSD) and considering limitations of pupillometry.

∗∗This technology is not intended as a substitute for invasive ICP monitoring.

### 3.30 Suggestion 30

We suggest that a MFM specialist should be immediately involved in the care of pregnant women with severe isolated TBI to assess fetal viability and well-being, as well as the most appropriate form of fetal monitoring (i.e., ultrasonography, fetal heart rate monitoring) (agreement 98.2%, strong suggestion).

### 3.31 Suggestion 31

We suggest that intermittent pneumatic compression devices (if available and feasible) should be utilized as soon as possible for deep vein thrombosis (DVT) prophylaxis in pregnant women with severe isolated TBI (agreement 98.2%, strong suggestion).

### 3.32 Suggestion 32

We suggest considering the initiation of pharmacologic DVT prophylaxis within 24-72 hours of injury following stable head imaging in pregnant women with severe isolated TBI. In this regard, a multidisciplinary consultation (e.g., neurosurgeon, MFM specialist, etc.) is advisable (agreement 94.7%, strong suggestion).

### 3.33 Suggestion 33

In pregnant women, when the uterus is visible above the umbilicus or known gestational age of 20-24 weeks, with severe isolated TBI suffering a cardiac arrest, we suggest that perimortem caesarean section (also known as resuscitative hysterotomy) should be considered as soon as possible (ideally, within no more than 4-5 minutes) (agreement 95.6%, strong suggestion).

### 3.34 Suggestion 34

For difficult and controversial situations in the management of pregnant women with severe isolated TBI (e.g., timing of emergency neurosurgery, timing/modality of delivery, abortion, maternal brain death and pregnancy progression, etc.), we suggest a multidisciplinary approach, with the possible aid of a clinical ethicist, to optimize decision-making in this challenging scenario (agreement 99.1%, strong suggestion).

### 3.35 Suggestion 35

For difficult and controversial situations in the management of pregnant women with severe isolated TBI (e.g., timing of emergency neurosurgery, timing/modality of delivery, termination of pregnancy, maternal brain death and pregnancy progression, etc.), we suggest a clear communication strategy with the family or next of kin (agreement 99.1%, strong suggestion).

### 3.36 Suggestion 36

We suggest that specific hospital protocols, based on the best available evidence and literature, regarding the management of pregnant women with severe isolated TBI, should be encouraged and implemented to optimize maternal and fetal outcomes (agreement 98.2%, strong suggestion).

## 4 Discussion

### 4.1 Multi-disciplinarity

This multidisciplinary and international consensus working group reports a list of suggestions for the management of severe isolated TBI during pregnancy, which remains a rare and challenging scenario. For this reason, pregnant women with severe isolated TBI should be admitted to a hospital with continuous availability of neurosurgical, MFM, and neonatal facilities. This is deemed mandatory as the gestational age increases and the period in which the fetus is potentially viable is entered. In any case, it is desirable that, regardless of the gestational age, the pregnant woman with severe TBI is admitted to a center where all relevant specialties are present.

### 4.2 Specific hospital protocols

We encourage the creation of specific hospital protocols based on the best available evidence and literature. In our opinion, this is important to ensure that all relevant specialists involved in the management of these patients are made aware of the physiological changes associated with pregnancy. Furthermore, the creation of protocols and/or guidelines could allow clear designation of the leader and the members of the trauma team specific to the organizational characteristics of the various hospitals, in order to facilitate the care pathway. In cases of severe TBI during pregnancy, there are two patients involved, hence the prompt involvement of MFM to assess fetal viability and well-being as well as the most appropriate form of fetal monitoring (i.e., ultrasonography, fetal heart rate monitoring) is very important. These procedures require smooth coordination and collaboration with other members of the trauma team and should not interfere with resuscitation and care of the mother (Jain et al., 2015a).

### 4.3 Imaging

Brain CT scan, with or without intravenous contrast, should be utilized in the acute phase and/or in life-threatening situations, without delay for fear of fetal exposure to ionizing radiation/contrast agents and with the use of radiation-minimizing imaging protocols (standard of new CT scanners). This suggestion was widely proposed in this scenario and should be a standard of practice (Jain et al., 2015a; Di Filippo et al., 2022; Wiles et al., 2022; Malaiyandi et al., 2021). The practice of abdominal shielding is controversial. The improvement of imaging technology contributed to a reduction of maternal and fetal radiation exposure without documented harm to the fetus (Wiles et al., 2022; Malaiyandi et al., 2021; American College of Radiology, 2008; American Association of Physicists in Medicine, 2019). Moreover, during radiological examination, the main source of radiation dose to organs outside the imaging field is from the internal rather than the external scatter (Wiles et al., 2022; American Association of Physicists in Medicine, 2019). Shielding may also negatively affect the automatic exposure control for the reduction of radiation dose and image quality. Consequently, the practice of shielding can paradoxically increase patient radiation dose (American Association of Physicists in Medicine, 2019; British Institute of Radiology et al., 2020). Considering the above, we recommend against abdominal shielding when performing brain CT scan in pregnant women with severe isolated TBI. Reassuringly, this is consistent with the recently published 11th edition of the Advanced Trauma Life Support (ATLS®) course manual, where this practice is not reported (American College of Surgeons, 2025). Probably, it might be important to communicate the rationale of this approach to the family, to prevent that the of lack of shielding being perceived as a negligence. Outside the acute phase and in the absence of life-threatening situations, the utilization of non-contrast brain MRI should be encouraged rather than brain CT scan (Di Filippo et al., 2022; Wiles et al., 2022; Malaiyandi et al., 2021). Gadolinium-based contrast agents are generally not recommended during pregnancy due to a lack of robust safety data regarding fetal exposure (Wiles et al., 2022; Malaiyandi et al., 2021). Animal studies showed gadolinium-associated teratogenic effects, but the relevance on human fetal development is unclear (Malaiyandi et al., 2021). Its use should be considered when the diagnostic benefit outweighs the unknown risks, as also reported by different guidelines (- Committee Opinion No, 2017; The Royal College of Radiologists, 2019; - American College of Radiology, 2021). According to the above, we suggest avoiding gadolinium-based contrast agents in pregnancy, unless there is no alternative study, and delaying imaging until the post-partum period would result in direct harm to mother or fetus.

### 4.4 ICP monitoring

Regarding indications for invasive ICP monitoring, there is no reason to treat pregnant patients with severe isolated TBI differently from non-pregnant patients. Therefore, invasive ICP monitoring should follow standard recommendations (Stocchetti et al., 2014; Le Roux et al., 2014; American College of Surgeons and Trauma Quality Programs, 2024). Recently, the use of non-invasive methods (i.e., brain ultrasonography and automated pupillometry) for ICP monitoring has received much interest (Brasil et al., 2024; Robba et al., 2025). At the time of writing, this technology cannot be considered a substitute for invasive ICP monitoring. According to the recent B-ICONIC consensus on nICP monitoring when invasive systems are not available in the care of TBI patients, a multimodal strategy with at least two concordant nICP modalities was recommended along with clinical examination and brain CT scan (when available) to assist in ICP management (Robba et al., 2025). However, brain ultrasonography is an operator-dependent technique requiring training and experience (Robba et al., 2019). Furthermore, noninvasive methods are not considered standard of care.

Our suggestions predominantly refer to hospitals in high-income countries which presumably are able to invasively monitor ICP when indicated. We have introduced, in our consensus suggestions, the non-invasive methods as screening tools that can be useful (integrated with clinical examination and radiology) in providing additional guidance on the need for invasive ICP monitoring. Furthermore, while awaiting ICP monitor placement, these methods can help clinicians implement therapy to control IH. It should be kept in mind that the approach proposed by B-ICONIC requires further validation in daily clinical practice and, again, in the case of pregnancy.

Regarding ICP/CPP targets, arterial transducer zeroing and SABP/MAP targets in the absence of invasive ICP monitoring (such absence should not be frequent given that we are talking about severe TBI), there are no data in the literature that lead to management modifications with respect to non-pregnant women (Carney et al., 2017; Hawryluk et al., 2019; Thomas et al., 2015; Rossaint et al., 2023). Regarding hemodynamic targets (with and without ICP), it is the opinion of some panelists that the changes induced by pregnancy should also be considered. Pregnant women generally exhibit low blood pressure during the first two trimesters and a tendency toward hypertension during the third trimester (Godoy et al., 2022). During pregnancy we observe a widening of the cerebral autoregulatory range, with a decreased lower limit and an increased upper limit of autoregulation; these changes, which improve the cerebral tolerance to hypotensive/hypertensive episodes, are deranged in preeclampsia (Godoy et al., 2022). Considering the above, it is critical to underline the importance of a multidisciplinary approach for the management of severe TBI patients who are pregnant and the usefulness of combined multimodal neuromonitoring, including invasive (i.e., ICP and brain tissue oxygenation) and non-invasive (i.e., brain ultrasonography) tools, as well as continuous fetal monitoring (Godoy et al., 2022). Regarding arterial transducer zeroing, some panelists proposed using two transducers to monitor both uterine perfusion and CPP. Moreover, the “dual transducers” approach (i.e., one zeroed at the level of the tragus and the other at the level of the right atrium) has been recently proposed in the neurocritical setting to optimize arterial pressures and perfusion to all organs (Mikkonen et al., 2024). At the moment, it is a sophisticated and valuable addition for advanced centers.

### 4.5 Positioning the patient

The positioning of the head and neck in the setting of severe TBI is crucial. Independent of pregnancy, the head of the bed should be maintained at 30°-45° to facilitate brain venous drainage (Hawryluk et al., 2019; Picetti et al., 2023). The head should also be maintained in the midline to avoid compression of the neck veins (Hawryluk et al., 2019; Picetti et al., 2023). Moreover, approximately after mid-pregnancy, when the uterus is at or above the umbilicus, it is essential to position the pregnant woman in left lateral tilt to minimize negative hemodynamic effects related to uterine compression of the inferior vena cava (Lopez et al., 2023). In this case, careful positioning is crucial with protection and maintenance of spinal alignment (especially in case of spinal injury), head elevation, and avoidance of neck veins compression.

### 4.6 Optimal Hb level

Data regarding optimal Hb level in pregnant severe TBI patients are lacking. The Seattle International Severe Traumatic Brain Injury Consensus Conference (SIBICC) and the American College of Surgeons (ACS) guidelines recommend maintaining a Hb level >7 g/dL (Hawryluk et al., 2019; American College of Surgeons and Trauma Quality Programs, 2024). Different levels are recommended based on brain tissue oxygenation monitoring (American College of Surgeons and Trauma Quality Programs, 2024; Chesnut et al., 2020). The HEMOglobin Transfusion Threshold in Traumatic Brain Injury OptimizatioN (HEMOTION) trial showed that a liberal transfusion strategy [red blood cell (RBC) transfusion in case of Hb ≤ 10 g/dL] did not reduce the risk of an unfavorable neurologic outcome at six months when compared to a restrictive transfusion strategy (RBC transfusion in case of Hb ≤ 7 g/dL) (Turgeon et al., 2024). In the Transfusion Strategies in Acute Brain Injured Patients (TRAIN) trial, patients with acute brain injury (TBI, subarachnoid hemorrhage, and intracerebral hemorrhage) and anemia randomized to a liberal transfusion strategy (RBC transfusion in case of Hb < 9 g/dL) were less likely to have an unfavorable neurological outcome than those randomized to a restrictive strategy (RBC transfusion in case of Hb < 7 g/dL) (Taccone et al., 2024). During pregnancy, a physiological anemia is observed, with Hb values around 12 g/dL (Godoy et al., 2022). Considering the above, we suggest that pregnant women with severe isolated TBI not adopt a different transfusion threshold or target hemoglobin level compared to non-pregnant female severe TBI patients (currently between 7 and 9 g/dL). Transfusion decisions should be based on the pregnant patient's cardiorespiratory status, and on data from advanced neuromonitoring (i.e., brain tissue oxygenation) (Chesnut et al., 2020) and fetal monitoring.

### 4.7 PaO_2_ and PaCO_2_ levels

The European Society of Intensive Care Medicine (ESICM) guidelines recommend maintaining of a PaO_2_ of 80-120 mmHg in acute brain injury patients (Robba et al., 2020). The ACS guidelines recommend values between 80 and 100 mmHg (American College of Surgeons and Trauma Quality Programs, 2024). During pregnancy, normal PaO_2_ values are between 101 and 106 mmHg (Hegewald and Crapo, 2011). Therefore, we suggest that in pregnant women with severe isolated TBI, the PaO_2_ should be maintained at 80-120 mmHg (adjusted for altitude). Higher levels could be utilized according to advanced neuromonitoring (i.e., brain tissue oxygenation) (Chesnut et al., 2020) and fetal monitoring. During pregnancy, a state of “physiological” respiratory alkalosis is reported, with PaCO_2_ values ranging from 28 to 29 mmHg in the first trimester to 26-30 mmHg in the third trimester (Hegewald and Crapo, 2011). Considering the potential harm of hypocapnia on the injured brain (Coles et al., 2002), values < 32 mmHg are not recommended in the absence of brain tissue oxygenation monitoring (Hawryluk et al., 2019; Chesnut et al., 2020). No evidence of the effects of PaCO_2_ levels between 26 and 30 mmHg on the injured brain of pregnant women with severe TBI is reported. Considering the above, we suggest that the optimal PaCO_2_ target, in this setting, should be based on neurological (e.g., ICP and brain tissue oxygenation monitoring, brain ultrasonography) and fetal monitoring. In the absence of these monitoring, we are unable to recommend the target of PaCO_2_ in this scenario.

### 4.8 Coagulopathy

Coagulopathy, related to TBI or medications, is reported frequently in severe TBI patients and is associated with the progression of intracranial mass lesions and, in some cases, with unfavorable neurological outcome (Yuan et al., 2016; Böhm et al., 2021; Maegele, 2021). Rapid correction of coagulopathy is very important, especially for patients requiring urgent neurosurgical procedures. Coagulation parameters suggested for neurosurgery are: PLT count >75.000 or 100.000/mm^3^ and PT/aPTT <1.5 the normal control values (American College of Surgeons and Trauma Quality Programs, 2024; Rossaint et al., 2023). Point-of-care tests, such as TEG and ROTEM, may be useful to individualize therapy in this setting, particularly when antiplatelet drugs and/or direct oral anticoagulants are utilized and/or to elucidate coagulopathy in patients who are unable to provide a history and taking medications which are not identifiable on conventional coagulation tests (Frontera et al., 2016). Pregnancy is generally associated with a hypercoagulable state (Godoy et al., 2022; Costantine, 2014), although a reduction in the PLT count (rarely <100,000/mm^3^) is observed during the third trimester (Reese et al., 2018). In this regard, some panelists emphasized the importance of not overcorrecting coagulation deficiencies in this setting. Although more studies are necessary to generate evidence about clinical outcomes, the utilization of viscoelastic tests (TEG and ROTEM) is also increasing in the setting of pregnancy, especially for assessing the risk of thrombosis and hemorrhage in different pathological conditions (Othman et al., 2019; Amgalan et al., 2020).

### 4.9 Venous thromboprophylaxis

Considering the risk of venous thromboembolism (VTE) in severe TBI, other guidelines recommend the start of pharmacological thromboprophylaxis 24-48 hours (American College of Surgeons and Trauma Quality Programs, 2024) or 24-72 hours after hospital admission (Ley et al., 2020; Yorkgitis et al., 2022). In this scenario, clinicians should balance the risk of post-traumatic intracerebral hemorrhage progression against that of VTE (American College of Surgeons and Trauma Quality Programs, 2024; Ley et al., 2020; Yorkgitis et al., 2022). Mechanical DVT prophylaxis should be applied as soon as possible after admission, in all TBI patients regardless of pharmacologic prophylaxis (American College of Surgeons and Trauma Quality Programs, 2024; Ley et al., 2020). Our suggestions are in accordance with the above. Pregnant patients classically present a state of progressive hypercoagulability which must be taken into consideration (Ley et al., 2020). In this regard, we consider a multidisciplinary consultation particularly useful for commencing pharmacological DVT prophylaxis in the most appropriate way.

### 4.10 Osmotherapy

Osmotherapy is a tier-1 recommended intervention for the management of IH in the SIBICC guidelines (Hawryluk et al., 2019). The serum sodium should be maintained <155 mEq/L and osmolality <320 mOsm/kg (Hawryluk et al., 2019). No human studies are available regarding the utilization of mannitol or hypertonic saline solutions during pregnancy (Godoy et al., 2022). For some panelists, the maximum serum sodium value proposed by the SIBICC could be dangerous for the fetus during pregnancy. When osmotherapy is used, excessive dehydration of the mother and fetus must be avoided. This can lead to placental malfunction and potential amniotic fluid reduction with consequences such as oligohydramnios, fetal growth restriction, or alterations on fetal monitoring (Hofmeyr and Gülmezoglu, 2002; Mulyani et al., 2021). Careful monitoring of the mother's volume status, aiming for the maintenance of normovolemia, can be integrated with fetal monitoring to avoid these complications.

### 4.11 Glycemic status

Glucose is a key nutritional substrate for the brain (Godoy et al., 2016). Both hypoglycemia and hyperglycemia are detrimental after severe TBI (Godoy et al., 2016). In this context, brain glucose demand is increased and hypoglycemia is very dangerous (Godoy et al., 2016; Vespa et al., 2012). For this reason, recent guidelines suggest maintaining glucose between 100 and 180 mg/dl allowing a state of “permissive” hyperglycemia (American College of Surgeons and Trauma Quality Programs, 2024). In case of gestational diabetes, the recommended glucose values are: 1) 70-95 mg/dL during fasting, 2) 110-140 mg/dL 1 h after meals, and 3) 100-120 mg/dL 2 h after meals (American Diabetes Association Professional Practice Committee, 2024). Hyperglycemia in pregnancy increases the risk of adverse pregnancy outcomes (e.g. preeclampsia, macrosomia, etc.) and neonatal complications (e.g., hypoglycemia, respiratory distress, etc.) (HAPO Study Cooperative Research Group et al., 2008; Sweeting et al., 2024). Data on the optimal serum glucose level in pregnant patients with severe TBI are lacking. Considering the above, we suggest the maintenance of serum glucose between 100 and 180 mg/dL in pregnant women with severe isolated TBI. Some panelists suggested that the upper value not exceed 140-150 mg/dL. However, others felt that this last approach could expose the mother's injured brain to detrimental hypoglycemic episodes. If this approach is adopted, very careful glucose monitoring is mandatory.

### 4.12 Tier-three therapies in refractory IH

Tier-three therapies, generally reserved for refractory IH, comprise: 1) secondary DC, mild hypothermia (35-36 °C), and barbiturate coma (Hawryluk et al., 2019; American College of Surgeons and Trauma Quality Programs, 2024). A recently published retrospective multicenter study, involving 408 severe TBI patients, showed secondary DC to be associated with improved mortality and neurological outcome compared to barbiturates (Picetti et al., 2025b). In this consensus, particular concerns regarding barbiturates and mild hypothermia were reported. The utilization of barbiturates is generally associated with maternal immunosuppression, hemodynamic instability, and fetal malformations (Leach and Zammit, 2020; Godoy et al., 2022). Only one case report, reporting the safety of pentobarbital coma during pregnancy in a patient suffering an intracerebral hemorrhage from a ruptured dural arteriovenous fistula, is available (Patel et al., 2022). Accordingly, we suggest that barbiturate coma should not be utilized, if possible, as third-tier therapy, in pregnant women with severe isolated TBI. It should be considered if alternative treatment options are ineffective, balancing maternal-fetal risk/benefit, and as salvage therapy for the mother in the absence of other alternatives. Therapeutic hypothermia (TH) can induce fetal bradycardia, requiring strict fetal heart monitoring (Godoy et al., 2022). Few case reports reporting the successful utilization of post-cardiac arrest TH in pregnancy are available (Rittenberger et al., 2008; Chauhan et al., 2012; Oguayo et al., 2015; Hogg et al., 2020). Despite the lack of robust data in this scenario, secondary DC seems to be the safer option during pregnancy (Godoy et al., 2022). We suggest, in pregnant women with severe isolated TBI at risk of IH/cerebral ischemia, the maintenance of the core body temperature between 36 and 37.5 °C as in non-pregnant women with severe TBI. In case of utilization of a targeted temperature management strategy for ICP control in pregnant women with severe isolated TBI, we suggest that the body core temperature should not be maintained a level <36 °C to avoid fetal bradycardia. Lower levels could be utilized cautiously accompanied by fetal monitoring.

### 4.13 Seizure prophylaxis

Seizure prophylaxis in the setting of severe TBI is a controversial issue. The SIBICC suggests considering anti-seizure medications for only 1 week after trauma in the absence of indications to continue (Hawryluk et al., 2019). The Neurocritical Care Society guidelines for seizure prophylaxis in adults hospitalized with moderate–severe TBI suggest that: 1) seizure prophylaxis or not could be used in moderate–severe TBI (weak recommendation, low quality of evidence), 2) levetiracetam should be used rather than phenytoin/fosphenytoin (weak recommendation, very low quality of evidence), and 3).

a short duration (≤7 days) should be used rather than a longer duration (>7 days) of use (Frontera et al., 2024). Awaiting data from adequately powered and performed studies, we suggest that seizure prophylaxis should be utilized in pregnant women with severe isolated TBI as in non-pregnant women with severe TBI. Moreover, antiepileptic drugs associated with known fetal toxicity (e.g., valproate, phenobarbital, phenytoin, etc.) should be avoided, particularly during the first trimester (Hope and Harris, 2023). Levetiracetam seems to have an acceptable safety profile during pregnancy (Hope and Harris, 2023). In our opinion, consultation with a neurologist could be particularly useful in this condition.

### 4.14 Resuscitative hysterotomy

Resuscitative hysterotomy (also known as perimortem caesarean section) is generally recommended for viable pregnancies (i.e., ≥23 weeks, or fundal height two or more fingerbreadths above the umbilicus) in case of a maternal cardiac arrest (Jain et al., 2015b). The procedure should be performed ideally within 4-5 minutes to optimize both maternal resuscitation and fetal salvage (American College of Surgeons, 2025; Burruss et al., 2025; Larson et al., 2024). According to some authors, the procedure should be performed when the uterus is visible above the umbilicus or the gestational age is confirmed to be ≥ 20 to 24 weeks (Burruss et al., 2025; Larson et al., 2024). This approach ensures a better chance of fetal and maternal survival (i.e., eliminating the fetal metabolic demands and the gravid uterus potentially causing aortocaval compression) (Burruss et al., 2025; Larson et al., 2024). After delivery, fetal and maternal resuscitation should be continuously performed (Larson et al., 2024). Considering the above, we have included 20-24 weeks of gestation (or the uterus visible above the umbilicus) as a potential indication. A fetus of 20-22 weeks can have a very low chance to survive but may have a hemodynamic impact during cardiopulmonary resuscitation. Resuscitative hysterotomy, in this case, ensures the best possible chance of survival for the mother. This condition is an emergency requiring rapid decision-making, such as some neurosurgical procedures. However, there are other situations where more time is available allowing for a multidisciplinary decision-making approach including a clinical ethicist (e.g., abortion, maternal brain death and pregnancy progression, etc.). Furthermore, for comatose patients, clear communication with family members should be mandatory for a shared care pathway.

In our suggestions, we often refer to brain tissue oxygenation monitoring as reported by the SIBICC (Chesnut et al., 2020). We believe this type of monitoring, if available, is important in situations like those mentioned in our consensus. At this time, we have decided not to make a specific suggestion regarding its placement because the available literature does not show a clear improvement in neurological outcomes associated with its use (Gouvêa et al., 2022; Payen et al., 2023). Furthermore, this is a form of “focal” neuromonitoring, and data interpretation depends on where the probe is placed inside the brain parenchyma (Hawryluk et al., 2016).

In Table 2 we report some of our suggestions with comments that relate to maternal-fetal physiology and discussion points from panelists.

**Table 2:** Consensus suggestions with comments related to maternal-fetal physiology and discussion with panelists.

Consensus Suggestion	Comments
-SABP ≥110 mmHg -MAP ≥80 mmHg -CPP 60-70 mmHg	-Consider pregnancy-associated changes -Low ABP in the first 2 trimester, high ABP in the third trimester -Widening of cerebral autoregulation range (better brain tolerance to hypotensive/hypertensive episodes) -Cerebral autoregulation is deranged in the case of preeclampsia -Possible usefulness of combined multimodal neuromonitoring and fetal monitoring
-Arterial transducer zeroing at the tragus	-“Dual transducers” approach (i.e., one zeroed at the level of the tragus and the other at the level of the right atrium) to optimize brain and placental perfusion
-PLTs >75.000/mm^3^ -PT/aPTT <1.5 the normal control values -POC tests	-Avoid overcorrection considering the hypercoagulable state associated with pregnancy
-Na <155 mEq/L -Osmolality <320 mOsm/kg	-Excessive dehydration of the mother and fetus can lead to placental malfunction -Careful monitoring of mother's volume status, aiming at the maintenance of normovolemia, integrated with fetal monitoring could be useful to avoid these complications
-Glucose 100-180 mg/dL	-Considering recommendations regarding gestational diabetes, the upper limit should not exceed 140-150 mg/dL -In case of a more strict glycemic control, very careful glucose monitoring is mandatory to avoid detrimental hypoglycemic episodes
-Third tier therapies	-Secondary DC seems the safer option respect to barbiturate coma and TH

Abbreviations: ABP = arterial blood pressure, SABP = systolic arterial blood pressure, MAP = mean arterial pressure, CPP = cerebral perfusion pressure, PLTs = platelets, PT = prothrombin time, aPTT = activated partial thromboplastin time, POC = point-of-care, Na = sodium, DC = decompressive craniectomy, TH = therapeutic hypothermia.

### 4.15 Limitations and notes on the use of the current consensus suggestions

This consensus process aimed to support clinicians' decision making in the management of a pregnant woman with severe isolated TBI. These suggestions have been created to assist a physician's clinical judgment, which is necessary to provide appropriate and personalized therapy. We decided to address this rare topic with a Delphi approach because for each of the statements there are no robust data from randomized controlled studies and the likelihood of such studies on this topic is low. Results from the consensus scoping review demonstrate that only case reports/series or very low-level evidence exist. Therefore, our statements are solely based on expert opinion. Pregnancy is a well-known exclusion criterion from clinical trials (Heller et al., 2025). Moreover, for some aspects, there is no strong evidence even in severe TBI without pregnancy. Therefore, we have tried to adapt to pregnant women -considering the physiological changes of pregnancy-what is applied in severe TBI patients in light of the always important Hippocratic principle “*Primum Non Nocere*”.

The adoption of a modified Delphi approach -involving experts from different countries and specialties worldwide and providing useful and practical suggestions for such a challenging clinical scenario-may seem less rigorous than evidence-based guidelines. However, as discussed, the evidence base in this scenario is low at best. The suggestions promulgated in this work do not represent a standard of practice and have no legal implications. They are suggested plans of care based on the best available evidence and the consensus of experts, but they do not exclude other approaches as being within the standard of practice. Ultimately, responsibility for treatment results rests with those directly engaged therein and not with the consensus group. These guidelines refer to the management of severe isolated TBI in pregnant women admitted to a hospital within a high-income country; in low-resource settings, these guidelines may need adaptation, particularly regarding invasive monitoring.

We are aware of the fact that, in maternal trauma care, the priority is the mother's well-being (Jain et al., 2015a; American College of Surgeons, 2025; Burruss et al., 2025). Mother's resuscitation and stabilization are the first steps, as her health directly impacts the fetus. After the mother is stabilized, attention is then given to the fetus. However, it is also true that not everything is always *black* or *white*. There are situations where the therapeutic choices made must only consider the mother (and only her) but also situations where it is important also to consider the fetus. If the mother is in a desperate condition, and the fetus is not viable, we are all confident in applying therapies without considering the possible negative effects on the fetus. However, there may be conditions where the mother is rather serious (but not extremely so) and where the therapeutic choices can also consider the development of the fetus (even if not viable at the time of the mother's head trauma). We believe that a multidisciplinary approach, with a mutually respectful collaboration between different medical specialties, is essential in order to offer maximum care to the mother in a time-efficient manner, independent of gestational age, while at the same time providing maximal care to the fetus.

## 5 Conclusions

This international and multidisciplinary consensus collaboration was established to provide practical suggestions to optimize the care of pregnant women with severe isolated TBI. This work represents the first structured harmonization of maternal–neurological priorities in severe TBI, bridging neurosurgical principles with fetal-centered care. In the future, more research should be conducted to improve clinical outcomes in this challenging condition.

## Declaration of competing interest

NM is part of the Editorial Board of Acta Neurochirugica and Brain&Spine, and European Editor of the Journal of Neurotrauma.
