# Supplementary material for: The management of severe isolated traumatic brain injury in pregnancy: A joint consensus statement from the European Association of Neurosurgical Societies (EANS) and the World Society of Emergency Surgery (WSES)
Source: Brain Spine. 2026 Feb 19;6:105971. doi: 10.1016/j.bas.2026.105971 (PMC13127189; doi:10.1016/j.bas.2026.105971)
Supplement: Multimedia component 1 [file mmc1.docx]

**LIST of PARTICIPANTS to the MULTIDISCIPLINARY CONSENSUS PANEL**

**NEUROSURGERY:**

1. Bizhan Aarabi (USA)
2. P. David Adelson (USA)
3. Rocco Armonda (USA)
4. Antonio Belli (UK)
5. Giacomo Bertolini (ITALY)
6. Alessandro Bertuccio (ITALY)
7. Riccardo Boccaletti (ITALY)
8. Sérgio Brasil (BRAZIL)
9. Andras Buki (SWEDEN)
10. Ana M. Castano-Leon (SPAIN)
11. Randall M. Chesnut (USA)
12. Andreas K. Demetriades (UK)
13. Bart Depreitere (BELGIUM)
14. Deepak Gupta (INDIA)
15. Gregory W.J. Hawryluk (USA)
16. Peter J. Hutchinson (UK)
17. Corrado Iaccarino (ITALY)
18. Alfonso Lagares (SPAIN)
19. Laura Lippa (ITALY)
20. Teemu Luoto (FINLAND)
21. Ivo Kehayov (BULGARIA)
22. Sam Klein (BELGIUM)
23. Angelos Kolias (UK)
24. Tommi K. Korhonen (FINLAND)
25. Nicolò Marchesini (ITALY)
26. Niklas Marklund (SWEDEN)
27. David O. Okonkwo (USA)
28. Wellingson Paiva (BRAZIL)
29. Marios Papadopoulos (UK)
30. Aurelia Peraud (GERMANY)
31. Ondra Petr (AUSTRIA)
32. Daniel Pinggera (AUSTRIA)
33. Jussi P. Posti (FINLAND)
34. Elham Rostami (SWEDEN)
35. Andres M. Rubiano (COLOMBIA)
36. Franco Servadei (ITALY)
37. Ranjit D. Singh (NETHERLANDS)
38. Nicole A. Terpolilli (GERMANY)
39. Péter J. Tóth (HUNGARY)
40. Parmenion Tsitsopoulos (GREECE)
41. Alex Valadka (USA)
42. Thomas A. van Essen (NETHERLANDS)
43. Alexander Younsi (GERMANY)
44. Gianluigi Zona (ITALY)

**ACUTE CARE SURGEONS**

1. Fikri M. Abu-Zidan (UAE)
2. Luca Ansaloni (ITALY)
3. Goran Augustin (CROATIA)
4. Zsolt J. Balogh (AUSTRALIA)
5. Walter L. Biffl (USA)
6. Fausto Catena (ITALY)
7. Mircea Chirica (FRANCE)
8. Federico Coccolini (ITALY)
9. Raul Coimbra (USA)
10. Belinda De Simone (ITALY)
11. Areg Grigorian (USA)
12. Kenji Inaba (USA)
13. Andrew W. Kirkpatrick (CANADA)
14. Matthew J. Martin (USA)
15. Jeffry Nahmias (USA)
16. Lena M. Napolitano (USA)
17. Sandro Rizoli (QATAR)
18. Frederick B. Rogers (USA)
19. Oreste Romeo (USA)
20. Philip F. Stahel (USA)
21. Christine T. Trankiem (USA)

**INTENSIVISTS/NEUROINTENSIVISTS/ANESTHESIOLOGISTS**

1. Rafael Badenes (SPAIN)
2. Daniele Bellantonio (ITALY)
3. Pierre Bouzat (FRANCE)
4. Etrusca Brogi (ITALY)
5. Anselmo Caricato (ITALY)
6. Carlo Alberto Castioni (ITALY)
7. Carlo Coniglio (ITALY)
8. Cristian Deana (ITALY)
9. Maria Grazia Frigo (ITALY)
10. Alberto Giannini (ITALY)
11. Daniel A. Godoy (ARGENTINA)
12. Paolo Gritti (ITALY)
13. Raimund Helbok (AUSTRIA)
14. Christos Lazaridis (USA)
15. Deepa Malaiyandi (USA)
16. Geert Meyfroidt (BELGIUM)
17. Marina Munari (ITALY)
18. Rita Pini (ITALY)
19. Edoardo Picetti (ITALY)
20. Hemanshu Prabhakar (INDIA)
21. Frank Rasulo (ITALY)
22. Chiara Robba (ITALY) - (non-voting methodologist)
23. Carla Bittencourt Rynkowski (BRAZIL)
24. Aarti Sarwal (USA)
25. Deepak Sharma (USA)
26. Lori Shutter (USA)
27. Jose I. Suarez (USA)
28. Fabio S. Taccone (BELGIUM)
29. Andrew A. Udy (AUSTRALIA)
30. Albert J. Varon (USA)
31. Monica S. Vavilala (USA)
32. Marco Vergano (ITALY)
33. Walter Videtta (ARGENTINA)
34. Tommaso Zoerle (ITALY)

**EMERGENCY PHYSICIANS**

1. Alessandro Cipriano (ITALY)
2. Zaffer Qasim (USA)
3. Marcello Zinelli (ITALY)

**MATERNAL-FETAL MEDICINE:**

1. Roberto Berretta (ITALY)
2. CeCe Cheng (USA)
3. Dubravko Habek (CROATIA)
4. Arundhathi Jeyabalan (USA)
5. Antonio Ragusa (ITALY)

**NEURORADIOLOGISTS/RADIOLOGISTS**

1. Christian Abel (AUSTRALIA)
2. Davide Cerasti (ITALY)
3. Enrico Fainardi (ITALY)
4. Savvas Nicolaou (USA)
5. Louis Smith (USA)

**NEUROLOGISTS**

1. Alessandro Pezzini (ITALY)

**NEONATOLOGISTS**

1. Enrico Cocchi (ITALY)
2. Luigi Orfeo (ITALY)
