# Supplementary material for: The management of severe isolated traumatic brain injury in pregnancy: A joint consensus statement from the European Association of Neurosurgical Societies (EANS) and the World Society of Emergency Surgery (WSES)
Source: Brain Spine. 2026 Feb 19;6:105971. doi: 10.1016/j.bas.2026.105971 (PMC13127189; doi:10.1016/j.bas.2026.105971)
Supplement: Multimedia component 2 [file mmc2.docx]

PICO

P: Adult pregnant patients with severe TBI admitted to the intensive care unit

I: Use of strategies for the management of severe TBI and for control of intracranial hypertension

C: Patients not treated for intracranial hypertension

O: mortality (any timepoints), neurological outcome, improvement of ICP and cerebral/ neuromonitoring parameters

String for search:

(“brain trauma”[MeSH] OR “traumatic brain injury”[All Fields] OR “head trauma”[All Fields] OR “head injury”[All Fields])OR”brain edema”[All Fields] AND (“Pregnancy” [All Fields])OR” pregnant” [All Fields] AND (“intracranial hypertension”[Mesh] OR “Cerebral perfusion pressure”[MeSH] OR “decompressive craniectomy”[MeSH] OR “tier therapies”[Mesh] OR “mannitol”[MeSH] OR “hypertonic saline”[MeSH] OR “hypotermia”[All Fields] OR “barbiturates”[All Fields]) AND (“outcome” [MeSH Terms] OR “mortality” OR “neurological outcome”) AND(“humans”[MeSH Terms] AND English[lang]) NOT (child* OR infant* OR pediatrics).
